# Editorial: Discrete emotions in environmental decision-making

**DOI:** 10.3389/fpsyg.2023.1272343

**Published:** 2023-09-05

**Authors:** Eugene Y. Chan, Katharine Howie, Felix Septianto

**Affiliations:** ^1^Ted Rogers School of Management, Toronto Metropolitan University, Toronto, ON, Canada; ^2^College of Business and Economic Development, University of Southern Mississippi, Hattiesburg, MS, United States; ^3^School of Business, The University of Queensland, Brisbane, QLD, Australia

**Keywords:** Editorial, environmental decision-making, sustainability, emotions, psychology

Climate change is a pressing global issue that demands immediate attention due to its far-reaching implications not just for the environment but also human well-being. As the Earth's climate continues to warm, extreme weather events, rising sea levels, and habitat destruction threaten ecosystems and livelihoods. The significance of climate change lies in its potential to disrupt the delicate balance of life on our planet. People play a crucial role in addressing this crisis by making informed environmental decisions (White et al., 2019). From adopting sustainable practices in daily life, such as reducing carbon footprints and conserving energy, to supporting eco-friendly policies and technologies, individuals can collectively drive positive change. Without the support of individuals, global warming and the associated negative externalities are expected to continue or intensify.

Consumers' pro-environment decisions are likely influenced by a combination of factors. Environmental awareness, personal values, perceptions of self-efficacy, and social norms play a crucial role in the climate related choices consumers make (Du Bray et al., 2019; Gregersen et al., 2020). Purchasing green products is one of many climate-conscious decisions consumers can make, and the success of environmentally friendly products is shaped by a diverse range of factors (Barbarossa and De Pelsmacker, 2016). Taken together, countless individual and situational attributes interact to shape an individual's attitudes and environmental choices. Empirical research from highly diverse disciplines have sought to provide insights into this consequential and complex domain. As research continues to evolve, a more comprehensive understanding of these factors informs strategies to encourage environmentally-friendly consumer behavior.

For the past few decades, emotion research has demonstrated how emotional valence differentially influences individuals' decision making (Peters et al., 2006). However, there is still very limited understanding regarding how discrete emotions can influence environmental behavior, potentially due to the complex nature of climate change (Pihkala, 2022). Understanding the role of emotions is vital in this setting as research has demonstrated strong predictive power for outcomes like climate mitigation (Xie et al., 2019), preference for energy technologies (Jobin and Siegrist, 2018), and support for policy (Smith and Leiserowitz, 2014). Environmental decisions often involve complex and multifaceted issues with long-term consequences (Larson et al., 2015), and people's emotional responses can heavily influence their attitudes and actions (Davidson and Kecinski, 2022). Positive emotions like empathy and concern for nature can motivate individuals to engage in pro-environmental behaviors, such as recycling or supporting conservation efforts (Berenguer, 2007). Conversely, negative emotions like fear or denial can hinder environmental action or lead to unsustainable practices (Bostrom et al., 2018).

This Research Topic of *Frontiers* recognizes the emotional underpinnings of environmental decision-making, policymakers, educators, and advocates can tailor their messages and strategies to appeal to people's emotions in ways that inspire positive environmental actions. The first paper in our Research Topic, Shipley et al. focus on two discrete emotions—pride and guilt. The authors how place attachment influence these two discrete emotions, thereby influencing pro-environmental behavior. Then, Sanford et al. focus on social media—specifically, the authors examine Twitter “tweets” and the emotional content they contain as they relate to environmental awareness. The concern for protecting the planet continues to be examined with Seibt et al. addressing how communal sharing relationships evoke an emotion termed “kama muta,” thereby expanding an understanding of how sympathy, compassion, and care influence environmental decision-making. Myers et al. then shift focus to communication strategies. Specifically, the authors examine how emotions regarding climate change information (not climate change more generally) influence their support for policy measures, focusing on the five emotions of guilt, anger, hope, fear, and sadness. Recognizing the global scope and complexity of climate concerns, Böhm et al. use Appraisal Theory to examine cross-cultural differences in emotional reactions to climate change and climate related actions. Zhang et al. also bring an international scope studying how adolescents' happiness may influence their willingness to protect the environment using data from eight countries. Our penultimate article, Geiger et al. use meta-analysis to investigate the effectiveness of hope in promoting sustainable decisions.

Future research on the role of individuals' emotions in environmental decision-making should further explore how emotional responses vary across different environmental issues and contexts beyond culture (Böhm et al.) and age (Zhang et al.). Investigating whether emotions differ in intensity and directionality depending on the type of environmental concern (e.g., climate change, deforestation, and pollution) and the geographic, cultural, or socio-economic context in which individuals are situated can provide valuable insights for tailoring communication and policy approaches.

Understanding the nuanced emotional landscape surrounding diverse environmental challenges can inform targeted interventions that resonate with individuals' emotional realities, fostering more profound connections to nature and driving meaningful pro-environmental actions. Additionally, research could delve into the interplay between emotions and cognitive processes in online environments as communication these days are largely done using social media (Sanford et al.). Ultimately, these investigations can empower policymakers, educators, and activists to effectively harness emotions as a force for positive environmental change. Indeed, recognizing emotions not toward climate change itself but toward communication (Myers et al.) will further expand how emotions play a role in sustainable decision-making. Policy-makers can design incentives and rewards that trigger positive emotions for adopting eco-friendly behaviors, reinforcing the link between personal well-being and environmental responsibility. Finally, new methods such as meta-analyses (Geiger et al.) can better assess the effectiveness of strategies and interventions beyond traditional psychological methods of surveys and experiments.

Indeed, policy-makers can strategically leverage people's emotions to promote environmentally friendly choices and decisions by employing targeted communication and policy interventions, as Myers et al. has shown. By crafting compelling narratives that evoke empathy and concern for the environment, policy makers can raise awareness about pressing environmental issues and their potential impact on communities and future generations. Utilizing positive emotional appeals, such as hope and optimism (Myers et al.), or guilt, pride and even sympathy (Shipley et al.; Seibt et al.) policy makers can highlight success stories and the transformative potential of sustainable practices, inspiring individuals to take action. By tapping into the emotional dimensions of decision-making, policy makers can foster a sense of shared responsibility and collective action, empowering individuals to become agents of positive environmental change.

## Author contributions

EC: Writing—original draft, Writing—review and editing. KH: Writing—original draft, Writing—review and editing. FS: Writing—original draft, Writing—review and editing.
